# The Use of Social Media for Dissemination of Research Evidence to Health and Social Care Practitioners: Protocol for a Systematic Review

**DOI:** 10.2196/45684

**Published:** 2023-05-12

**Authors:** Sarah F Roberts-Lewis, Helen A Baxter, Gill Mein, Sophia Quirke-McFarlane, Fiona J Leggat, Hannah M Garner, Martha Powell, Sarah White, Lindsay Bearne

**Affiliations:** 1 Population Health Research Institute St George's University of London London United Kingdom; 2 Bristol Population Health Science Institute University of Bristol Bristol United Kingdom; 3 National Institute of Health and Care Research London United Kingdom; 4 Faculty of Health, Social Care and Education St George's University of London London United Kingdom; 5 School of Psychology University of Surrey Guildford United Kingdom; 6 Department of Physiotherapy St George’s University Hospitals NHS Foundation Trust London United Kingdom

**Keywords:** dissemination, health care, podcast, practitioners, research evidence, social care, social media, social networking, Twitter, videos

## Abstract

**Background:**

Effective dissemination of research to health and social care practitioners enhances clinical practice and evidence-based care. Social media use has potential to facilitate dissemination to busy practitioners.

**Objective:**

This is a protocol for a systematic review that will quantitatively synthesize evidence of the effectiveness of social media, compared with no social media, for dissemination of research evidence to health and social care practitioners. Social media platforms, formats, and sharing mechanisms used for effective dissemination of research evidence will also be identified and compared.

**Methods:**

Electronic database searches (MEDLINE, PsycINFO, CINAHL, ERIC, LISTA, and OpenGrey) will be conducted from January 1, 2010, to January 10, 2023, for studies published in English. Randomized, nonrandomized, pre-post study designs or case studies evaluating the effect of social media on dissemination of research evidence to postregistration health and social care practitioners will be included. Studies that do not involve social media or dissemination or those that evaluate dissemination of nonresearch information (eg, multisource educational materials) to students or members of the public only, or without quantitative data on outcomes of interest, will be excluded. Screening will be carried out by 2 independent reviewers. Data extraction and quality assessment, using either the Cochrane tool for assessing risk of bias or the Newcastle-Ottawa Scale, will be completed by 2 independent reviewers. Outcomes of interest will be reported in 4 domains (reach, engagement, dissemination, and impact). Data synthesis will include quantitative comparisons using narrative text, tables, and figures. A meta-analysis of standardized pooled effects will be undertaken, and subgroup analyses will be applied, if appropriate.

**Results:**

Searches and screening will be completed by the end of May 2023. Data extraction and analyses will be completed by the end of July 2023, after which findings will be synthesized and reported by the end of October 2023.

**Conclusions:**

This systematic review will summarize the evidence for the effectiveness of social media for the dissemination of research evidence to health and social care practitioners. The limitations of the evidence may include multiple outcomes or methodological heterogeneity that limit meta-analyses, potential risk of bias in included studies, and potential publication bias. The limitations of the study design may include potential insensitivity of the electronic database search strategy. The findings from this review will inform the dissemination practice of health and care research.

**Trial Registration:**

PROSPERO CRD42022378793; https://www.crd.york.ac.uk/prospero/display_record.php?RecordID=378793

**International Registered Report Identifier (IRRID):**

DERR1-10.2196/45684

## Introduction

### Background

Health and social care researchers aim for their research findings to be accessible and useful for practitioners to help them deliver best, evidence-based clinical care and improve patient outcomes [1-4]. Thus, as part of continuing professional development, practitioners need to access relevant high-quality research evidence [5,6]. Research evidence is defined as information provided by a research study; information may derive from original studies (primary research), reviews (secondary research), or evidence-based guidelines [6]. Effective strategies are required to deliver relevant research evidence to practitioners [2,7,8] because dissemination of research evidence without delay is recommended to maximize its benefits [9,10].

Dissemination is defined as active approaches that use specific channels and planned strategies to get research evidence to a specific audience (who can make use of it and enact research benefits) [10]. Challenges of dissemination to practitioners include organizational barriers, limited professional opportunities, time constraints, and accessing relevant articles within the exponentially increasing volume of research evidence produced each year [8,11-13]. Social media has potential to overcome some of the barriers to dissemination [7,8].

Social media is defined as a collection of web-based platforms that allow the creation and exchange of user-generated content [14]. Open social media are accessible to anyone; closed social media groups have eligibility parameters that limit user participation [15]. The number of people who use social media globally has risen from millions to billions in the last 20 years; its advantages include not being limited in time and space [7,16]. Thus, professional use of social media by health and social care practitioners represents an opportunity for effective dissemination of research evidence [15]. Indeed, social media is increasingly used to disseminate health and social care research [8,15,17-21].

Social media use in health care has been studied and reviewed extensively [22-28]. Reviews concur about benefits and risks of social media for reputation, communication, information sharing, and public health messages. For health care professionals, social media has also been used effectively for education and day-to-day communications [25,29,30].

The existing reviews of social media for dissemination of health care research have narratively synthesized benefits and risks, described similarities, differences, and qualitative experiences, or provided commentaries on the mechanisms and potential uses of social media for dissemination [15,25,26,31]. However, they are heterogeneous in terms of study design and perspectives [15,26,31]. In 2018, a review of reviews about effective uses of social media in public health and medicine included little evidence concerning dissemination [25]. In 2020, a review highlighted 4 research dissemination case studies [26]. An unpublished preprint review, which identified 4 randomized controlled trials and 37 quantitative or descriptive studies, identified Twitter as a prominent platform for dissemination [31]. A subsequent review in 2022 highlighted the communication mechanisms and potential of social media for both 1-way knowledge mobilization (ie, dissemination) and 2-way knowledge mobilization (ie, more complex multidirectional integrated knowledge translation involving collaborative interactions between researchers and practitioners) [15]. The existing reviews are health care focused; no reviews have investigated dissemination to social care practitioners.

Although quantitative reports and comparisons of social media for dissemination of research evidence in health care are emerging [18-20,32], it is not yet clear how consistent or robust their findings are. To date, no quantitative synthesis or meta-analysis has been undertaken to investigate the effectiveness of open social media for dissemination of research evidence to health and social care practitioners.

### Objective

The objective of this systematic review is to quantitatively synthesize evidence of the effectiveness of social media, compared to no social media, for dissemination of research evidence to health and social care practitioners. The social media platforms, formats, and sharing mechanisms used for effective dissemination of research evidence will also be identified and compared.

The specific research questions are as follows: (1) how effective is open social media for dissemination of research evidence for health and social care practitioners, compared to no social media input? (2) Which social media platforms, formats, and sharing mechanisms are used for dissemination of research evidence for health and social care practitioners? (3) What is the comparative effectiveness for dissemination of research evidence to health and social care practitioners between different social media platforms (eg, Facebook vs Twitter), different formats of social media posts (eg, text vs infographic vs video), and different social media–sharing mechanisms (eg, site-wide shares vs special interest groups vs live social media events vs influencer endorsements)

## Methods

### Design

The inception and design of this systematic review, of articles available from January 1, 2010, to January 10, 2023, incorporated patient and public involvement via consultations with a stakeholder group. This protocol follows PRISMA-P (Preferred Reporting Items for Systematic Reviews and Meta-Analyses) guidelines [33]. The protocol has been registered with the International Prospective Register of Systematic Reviews (PROSPERO; registration number: CRD42022378793).

### Eligibility

Table 1 summarizes the population, intervention, comparisons, and outcomes of interest. The target population will be practitioners, defined as health and social care professionals, including, but not limited to, some of the largest groups of registered practitioners in the United Kingdom; that is, nurses, doctors, social workers, midwives, pharmacists, physiotherapists, occupational therapists, radiographers, and paramedics [34-38]. We will include both individual professions and collective groups of practitioners.

**Table 1:** Definitions of participants, intervention, comparisons, and outcomes. The numbers in square brackets denote prioritization for analyses if more than one outcome is reported in a single study, according to the most reported outcomes. Prioritization will ensure outcomes are only represented once per study in quantitative synthesis and meta-analysis.

Term		Definition
**Participants**		
	Practitioners	Postregistration health and social care professionals, either as groups incorporating a range of disciplines (eg, workers at hospitals and other health and social care settings, clinicians and health and social care professionals) or as individual professions (eg, the largest registries of UK practitioners include 758,303 nurses and midwives [34], 355,250 doctors [35], 91,191 social workers [36], 79,628 pharmacists and pharmacy technicians [37], 60,783 physiotherapists [38], 41,732 occupational therapists [38], 39,497 radiographers [38], and 33,219 paramedics [38], and other disciplines. Similar profiles of common health and social care professions were expected in other geographical locations).
**Intervention**		
	Research evidence	Human, health and social care–related research, originating from published, peer-reviewed journal articles, that could be relevant for practitioners. Information posted on social media might be a direct link to these research articles, abstracts, or repackaged information from a single peer-reviewed research article source; eg, summaries such as microblogs, blogs, press articles, conference presentations or posters, infographics, or educational videos.
	Social media	Open social media platforms, including, but not limited to, social networking and media-sharing sites and apps, that allow any user to make a one-to-many post and to interact by responding to posts. The largest of these social media sites in 2018 in terms of active users included Facebook (2.26 billion), YouTube (1.90 billion), WhatsApp (1.33 billion), Instagram (1 billion), WeChat (1 billion), Tumblr (624 million), TikTok (500 million), Reddit (355 million), Twitter (329.5 million), and Pinterest (245.5 million) [16].
**Comparison**		
	Dissemination	Comparison of effectiveness of dissemination to practitioners using open social media vs no social media (between and within group comparisons) and comparison between social media platforms, sharing mechanisms, and formats.
**Outcomes**		
	Reach	Number of practitioners who access a research evidence–related social media post. Number of impressions [1], views [2], or accesses [3] of a research evidence–related social media post.
	Engagement	Number of positive responses (eg, likes) to a research evidence–related social media post. Number of interactions (eg, shares [1], comments [2], or fresh social media posts triggered [3]) with a research evidence–related social media post.
	Direct dissemination	Number of times an original research article is accessed (eg, link clicks [1] or HTML views [2]). Number of times an original research article is downloaded (eg, PDF download). Altmetric score of an original research article.
	Impact	Number of citations [1] of an original research article or [2] impact factor of journal. Measures of changes in thinking or practice of health and social care practitioners linked to a research evidence–related social media post targeted at health or social care practitioners.

The intervention will be open social media used for dissemination of research evidence. We define research evidence as information from peer-reviewed articles featuring empirical findings that have met the publication standards of their specialty. Evidence may take the form of an original research article, or a group of original research articles identified and synthesized systematically. In this review, only research evidence that is professionally relevant to health and social care practitioners will be considered; however, our definition includes open-access research evidence (and social media posts where practitioners are the target audience), even when other audiences, like the general public, also have access.

We define open social media as social networking and media-sharing sites or platforms that allow any user to make a one-to-many post and to interact by responding to posts. Our definition of social media differs from broader existing definitions of social media, such as that of Kaplan and Haelein [14], who consider social media as web-based applications that allow the creation and exchange of user-generated content. Our more focused definition was used to target social media with the greatest potential for knowledge mobilization between the different communities of research and clinical settings. The current literature in knowledge mobilization has highlighted the importance of open, 2-way interaction to create linkage between communities and facilitate knowledge sharing. In this context, dissemination of research is an active, relational, multidirectional, and collaborative process that interacts with the standpoint of those who access, share, discuss, and use it [39,40]. Thus, we do not include mass media press articles, wikis, and blogs with no, or limited, facility for user interactions, purely communication-based applications or closed, fee-paying, or invite-only social media groups. However, these will be considered if they are highlighted using open social networking or media sharing sites, used as part of onward information sharing, or are groups that can be freely joined by any interested user (eg, by following a link available on open social networking and media-sharing sites).

We define social media–sharing mechanisms as the ways in which research evidence might be featured or interacted with, including, but not limited to, open sharing to the entire forum, live social media events, influencer endorsement, and accessible special interest groups that can be joined or followed. We define social media formats as including a variety of media types, including, but not limited to, text, illustrative pictures, visual abstracts, infographics, videos, and podcasts.

The study inclusion and exclusion criteria are summarized in Textbox 1. Full-text, English-language studies will be included if they make quantitative comparison between dissemination of research evidence for health and social care practitioners using social media versus a controlled “no social media” condition (between group). Studies will also be included that quantify changes from baseline conditions to follow-up after an open social media campaign for dissemination of research evidence to health and social care practitioner (within group or case series data). Studies quantitatively comparing dissemination of research evidence for health and social care practitioners between different social media platforms, sharing mechanisms, and formats will also be included. Studies comparing topics of social media posts or research evidence will be excluded.

**Summary of inclusion and exclusion criteria.**

**Inclusion criteria:**

- Peer-reviewed journal articles, study types including randomized controlled trials, case-controlled comparisons nonrandomized comparisons, pre-post designs, cohort studies, and case reports.
- Articles published after 2010.
- English-language articles or translations in English available.
- Articles that quantitatively evaluate and compare open group social media in terms of reach, engagement, dissemination, and impact of research evidence for postregistration health and social care practitioners.

**Exclusion criteria:**

- Study types including protocols, reviews, opinion pieces, and conference abstracts.
- Articles published before 2010.
- Articles not available in English.
- Study populations not including health and social care practitioners (eg, no mention of practitioners, only focused on students, service users, patients, or the general public).
- Social media interventions used only for sharing non health and social care–related research topics or other sorts of information (eg, multisource clinical education delivery, day-to-day interpersonal communications, and organizational or administrative information), closed, private, or invite-only groups, professional identity and reputation purposes, recruitment, or posts without a social networking component (eg, a blog or press article without signposting on social media networking sites).
- Comparisons between information topics only (without other comparisons, eg, between social media and control conditions, or between social media sites, or types of media).
- Articles that do not provide sufficient quantitative data on outcomes of interest or those reporting only qualitative data.

### Information Sources

Searches will be carried out from January 1, 2010, to January 10, 2023. Preliminary searches revealed that there was unlikely to be relevant literature prior to 2010; whereas after 2010, reports of social media use in research dissemination increased. Searches will be carried out in electronic bibliographic databases including MEDLINE (Ovid), PsycINFO (Ovid), CINAHL plus (EBSCO), ERIC (EBSCO), LISTA, and OpenGrey. Search engines, including PubMed, elicit, and Google Scholar, will also be used for citation searches and reference harvesting. Bibliographic hand-searching of relevant systematic reviews and included articles will also be carried out. The search strategy is likely to be adequate to ameliorate selection and detection biases.

### Search Strategy

The search strategies were informed by previous review searches [15,31,41] (see the search terms in Table 2 and the example search strategies in Multimedia Appendix 1).

**Table 2:** Summary of key search terms.

Key term	Search terms
Practitioners	[Health, healthcare, health care, medical, hospital, social care] and [practitioner, professional, provider, staff, employee] or clinician Nurse, midwife, doctor, physician, social worker, pharmacist, physiotherapist, physical therapist, occupational therapist, radiographer, or paramedic
Intervention	Research evidence, information, research, data, knowledge, dissemination, sharing, knowledge mobilisation, knowledge translation.
Social media	Social media, social network, open network, media sharing, social web, or social software Facebook, YouTube, WhatsApp, Instagram, WeChat, Tumblr, TikTok, Reddit, Twitter, Pinterest, Flickr, Googl,, microblog, podcast, webcast, Tweet, or video sharing
Outcomes	Dissemination, reach, engagement, impact, quantitative, evaluation, comparison, access, views, impressions, likes, share, comments, posts, HTML views, altmetric, download, citation, in practice, knowledge use
Timeframe	January 2010-January 2023
Language	English
Subjects	Human

### Data Management

The search results will be exported to, and deduplicated in, Endnote online (Clarivate), then imported into Rayyan software (Rayyan Systems) for title, abstract, and full-text screening [42].

### Selection of Studies

Two reviewers (SRL and SQM) will independently screen titles and abstracts for eligibility. Potentially eligible studies will be obtained in full text and the eligibility criteria applied. Two of 5 independent reviewers (from a team including SRL, SQM, LB, HG, and FL) will screen all full-text articles against the eligibility criteria. If no consensus can be reached between reviewers, eligibility will be settled by a third reviewer (HB). Articles will be included only if they provide sufficient quantitative data to allow extrapolation of mean (SD) outcome data per social media post for 2 comparable groups. Reasons for article exclusion will be recorded according to the PRISMA recommendations [43].

### Data Extraction

The data will be extracted from included full-text articles by 2 of 5 reviewers (SRL and SQM/LB/HG/FL) independently using a data extraction form developed a priori and adapted from the previous relevant reviews [26,31] (see Multimedia Appendix 2). Consensus on extracted data accuracy will be achieved by discussion and settled by a third reviewer (HB) in the case of disagreement. Corresponding authors of eligible studies will be contacted to request additional data when required.

### Outcome Data

The quantitative outcomes will be grouped into 4 domains (reach, engagement, dissemination, and impact; Table 1) [27,44]. Outcome measures will be grouped by domain because a heterogeneous range of outcomes and study designs are characteristic of the social media literature [15,25]. There is no primary outcome because dissemination of research evidence via social media is a complex multidimensional behavior that cannot be distilled into a single outcome [25,27]. Different outcomes measuring the same concept will be combined for meta-analysis wherever appropriate. Where multiple outcomes reporting the same concept are reported in a single study, hierarchical outcome prioritization will be used, preferentially selecting the most frequently reported variables (see Table 1).

The difference between group means for each comparison group will be obtained, or the mean difference from baseline to follow-up in pre-post designs. To accompany means, SD and 95% CI will be extracted. Wherever possible missing means and SDs will be calculated from other reported statistics (eg, median and IQR, as per Cochrane Handbook instructions Section 5.6 and Chapter 6) [45]. For each outcome, data will be simplified by dividing by the number of social media posts, to yield comparable quantitative data per research evidence–related social media post.

### Risk of Bias and Quality Rating of Evidence

For randomized controlled trials, the Cochrane tool for assessing risk of bias (ROB-2) will be used [46]. For nonrandomized controlled trials, the Newcastle-Ottawa Scale (NOS) [47] will be used to assess risk of bias, as per 2019 Cochrane recommendations [45]. Both the ROB-2 and the NOS will be adapted to suit the types of studies selected in this systematic review because studies testing social media dissemination are unlikely to use typical participant grouping designs. The NOS was selected in preference to the latest Cochrane tool for assessing risk of bias in nonrandomized studies (ROBINS; consisting of ROBINS-I for intervention studies and ROBINS-E for exposure studies [48,49]). This was because the complexity of these tools will not allow adaptation to alternative study designs and 1 tool (ROBINS-E for exposure studies) is still in development.

ROB-2 consists of 34 items, divided into 6 scoring domains that differentiate between lower risk of bias and higher risk of bias [46]. The maximum rating of the NOS is 9, a score of 0-3 is considered high risk of bias, 4-6 considered a medium risk of bias, and 7-9 considered a low risk of bias (Multimedia Appendix 3) [47]. Risk of bias will be included in results tables and figures that describe each included study.

### Data Synthesis

Results will be summarized narratively with text, tables, and figures. Quantitative comparisons between studies and groups will include mean, SD, and simplified group means weighted per research evidence–related social media post.

Between, and within for pre-post designs, group differences, SDs, and standardized effect sizes (eg, Cohen *d* or Hedges *g*) will be used to compare effects between included studies. Effect sizes of ≥0.8 will be defined as large, ≥0.5 as moderate, and ≥0.2 as small [50].

For outcomes that are available in a sufficient number of studies, pooled effects will be tested in meta-analyses using RevMan (version 5.3; The Cochrane Collaboration). Heterogeneity will be tested using *I*^2^. To test pooled effects, a fixed-effects model will be used if *I*^2^ is less than 50% or a random-effects model will be used if *I*^2^ is greater than 50%. If a comparison has *I*^2^>75% it will be removed from meta-analysis and synthesized narratively instead. Subgroup analyses will be used for heterogeneous study groups. Publication bias will be assessed using funnel plots.

### Confidence in Cumulative Evidence

For each outcome, the uncertainty of the evidence base will be evaluated based on the grading of recommendations assessment, development, and evaluation approach [51]. This includes the following 5 domains: study limitations, imprecision, indirectness, inconsistency, and publication bias. The grading of recommendations assessment, development, and evaluation domains will be used to upgrade or downgrade evidence after initial assessment. Quality of evidence will be categorized as high, moderate, low, or very low [52,53].

## Results

**Figure 1:**
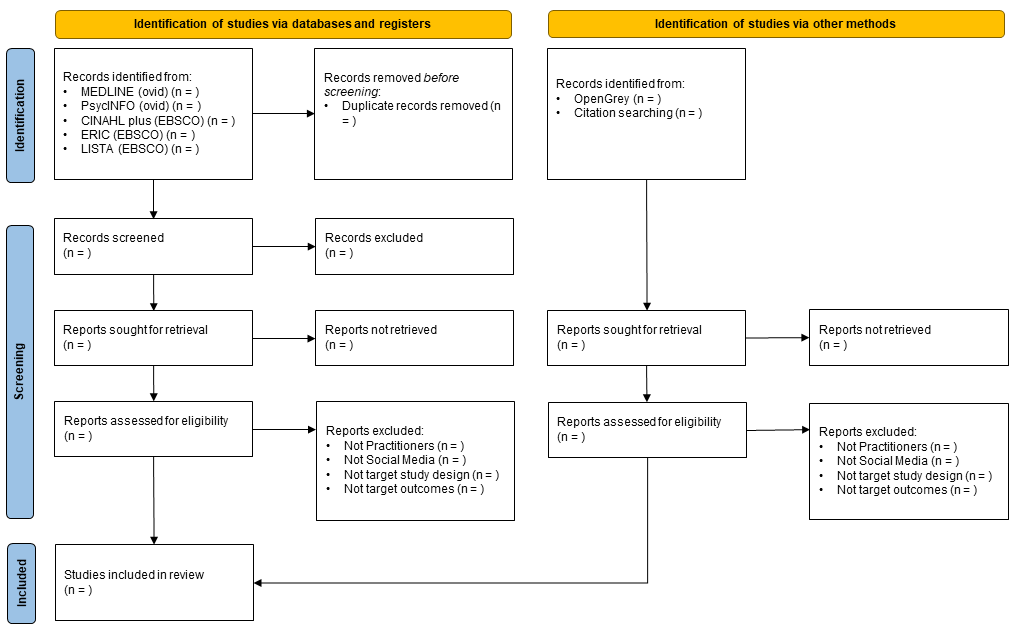
PRISMA (Preferred Reporting Items for Systematic Reviews and Meta-Analyses) flowchart.

Searches and screening will be completed by the end of May 2023 and recorded in a PRISMA flowchart (Figure 1) [43].

Data extraction and analyses will be completed by the end of July 2023, after which findings will be synthesized.

## Discussion

### Overview

We expect that this study will be completed by the end of October 2023. This review will synthesize evidence of the effectiveness of open social media for dissemination of research evidence to health and social care practitioners. We anticipate that we will find evidence that social media is effective, although there may be variation between its effect on the 4 outcome domains (reach, engagement, direct dissemination, and impact).

The previous reviews have highlighted the potential of social media for a variety of purposes in health care [15,25,26,31,54], although dissemination of research evidence currently accounts for only 1% of health research concerning social media [55]. Two existing reviews suggest that social media might be effective to improve direct dissemination and impact of research articles [31] and reach and engagement with clinical guidelines [54]; however, quantitative syntheses and meta-analyses were not performed.

Qualitative reviews have highlighted strategies that may enhance the dissemination of research evidence on social media [15,26,31,54]. These include enlisting people with expertise in social media at the planning stage, using professional social media marketing services and involving target users in content design. Use of multiple social media platforms is implicated, although Twitter is most often highlighted for professional use. Social media formats include using media that are accessible, relevant, useful, authentic, credible, and visually appealing to target users, a range of multimedia formats (including good quality images and infographics, text, videos, and podcasts, with images highlighted as being particularly important for social media reach and engagement). Social media–sharing mechanisms include involving key influencers and networks with the most connectedness in the field (such as organizational newsletters, journals, or individuals) to enhance social filtering, creating or enlisting existing web-based communities of practice, identifying and using key hashtags, posting at planned times (potentially including weekdays, weekday evenings, and weekends), posting regularly for a sustained campaign, and coordinating engagement events (such as journal clubs, question and answer sessions, or live interviews).

This systematic review will be the first to use quantitative, meta-analytical synthesis of evidence for the effectiveness of social media for research dissemination to practitioners. The limitations of the evidence may include multiple outcomes or methodological heterogeneity that limit meta-analyses, potential risk of bias in included studies, and potential publication bias. The limitations of the study design may include potential insensitivity of the electronic database search strategy to specific professional disciplines or social media platforms; however, bibliographic citation searching of included articles and relevant systematic reviews is likely to compensate for this. As social media are rapidly evolving, this systematic review will need to be repeated regularly to consider the impact of new and favored platforms and the search strategy revised to account for this.

The conclusions of this systematic review will inform the use of social media for the dissemination of research evidence to health and social care practitioners. This information will be important for researchers, research funders, and governmental bodies who have a remit to share their research evidence to inform practice and care. The findings of this review could be complemented by investigation of different practitioners’ attitudes and experiences of professional social media use so that targeted dissemination strategies could be developed. Future research directions include quantitative synthesis and meta-analysis of evidence for the effectiveness of closed social media groups for the dissemination of research evidence to health and social care practitioners. The interaction between closed and open social media may enhance knowledge sharing and engagement [15].

### Dissemination Plan

The findings from this systematic review will be disseminated through a peer-reviewed journal article, presentations at academic conferences and promoted using social media.

## Appendix

Multimedia Appendix 1 Search strategy.

Multimedia Appendix 2 Data extraction form.

Multimedia Appendix 3 Risk of Bias tools.

## Abbreviations

NOS: Newcastle-Ottawa Scale
PRISMA: Preferred Reporting Items for Systematic Reviews and Meta-Analyses
ROB-2: Cochrane tool for assessing risk of bias
ROBINS: Cochrane tool for assessing risk of bias in nonrandomized studies

## Data Availability

The data sets generated and analyzed during this study will be available from the corresponding author on reasonable request. The full search strategy is available in Multimedia Appendix 1.
