# Supplementary material for: The Use of Social Media for Dissemination of Research Evidence to Health and Social Care Practitioners: Protocol for a Systematic Review
Source: JMIR Res Protoc. 2023 May 12;12:e45684. doi: 10.2196/45684 (PMC10221530; doi:10.2196/45684)
Supplement: Multimedia Appendix 1 [file resprot_v12i1e45684_app1.docx]

Appendix 1

Search strategy examples

APA PsycInfo <1967 to November Week 1 2022> same used for Medline

1 health.mp. [mp=title, abstract, heading word, table of contents, key concepts, original title, tests & measures, mesh word] 882272

2 health care.mp. [mp=title, abstract, heading word, table of contents, key concepts, original title, tests & measures, mesh word] 238676

3 medical.mp. [mp=title, abstract, heading word, table of contents, key concepts, original title, tests & measures, mesh word] 265381

4 hospital.mp. [mp=title, abstract, heading word, table of contents, key concepts, original title, tests & measures, mesh word] 136537

5 social care.mp. [mp=title, abstract, heading word, table of contents, key concepts, original title, tests & measures, mesh word] 5249

6 healthcare.mp. [mp=title, abstract, heading word, table of contents, key concepts, original title, tests & measures, mesh word] 62451

7 1 or 2 or 3 or 4 or 5 or 6 1078387

8 practitioner*.mp. [mp=title, abstract, heading word, table of contents, key concepts, original title, tests & measures, mesh word] 105661

9 professional*.mp. [mp=title, abstract, heading word, table of contents, key concepts, original title, tests & measures, mesh word] 342832

10 provider*.mp. [mp=title, abstract, heading word, table of contents, key concepts, original title, tests & measures, mesh word] 79574

11 staff.mp. [mp=title, abstract, heading word, table of contents, key concepts, original title, tests & measures, mesh word] 91849

12 employee*.mp. [mp=title, abstract, heading word, table of contents, key concepts, original title, tests & measures, mesh word] 101253

13 8 or 9 or 10 or 11 or 12 621664

14 7 and 13 285653

15 clinician*.mp. [mp=title, abstract, heading word, table of contents, key concepts, original title, tests & measures, mesh word] 108773

16 midwif*.mp. [mp=title, abstract, heading word, table of contents, key concepts, original title, tests & measures, mesh word] 3036

17 nurse*.mp. [mp=title, abstract, heading word, table of contents, key concepts, original title, tests & measures, mesh word] 80312

18 doctor*.mp. [mp=title, abstract, heading word, table of contents, key concepts, original title, tests & measures, mesh word] 42704

19 physician*.mp. [mp=title, abstract, heading word, table of contents, key concepts, original title, tests & measures, mesh word] 85855

20 social worker*.mp. [mp=title, abstract, heading word, table of contents, key concepts, original title, tests & measures, mesh word] 29261

21 pharmacist*.mp. [mp=title, abstract, heading word, table of contents, key concepts, original title, tests & measures, mesh word] 3912

22 physiotherapist*.mp. [mp=title, abstract, heading word, table of contents, key concepts, original title, tests & measures, mesh word] 1564

23 physical therapist*.mp. [mp=title, abstract, heading word, table of contents, key concepts, original title, tests & measures, mesh word] 1790

24 occupational therapist*.mp. [mp=title, abstract, heading word, table of contents, key concepts, original title, tests & measures, mesh word] 6113

25 radiographer*.mp. [mp=title, abstract, heading word, table of contents, key concepts, original title, tests & measures, mesh word] 93

26 paramedic*.mp. [mp=title, abstract, heading word, table of contents, key concepts, original title, tests & measures, mesh word] 1141

27 14 or 15 or 16 or 17 or 18 or 19 or 20 or 21 or 22 or 23 or 24 or 25 or 26 511390

28 social media.mp. [mp=title, abstract, heading word, table of contents, key concepts, original title, tests & measures, mesh word] 22037

29 social network*.mp. [mp=title, abstract, heading word, table of contents, key concepts, original title, tests & measures, mesh word] 40358

30 open network*.mp. [mp=title, abstract, heading word, table of contents, key concepts, original title, tests & measures, mesh word] 42

31 media sharing.mp. [mp=title, abstract, heading word, table of contents, key concepts, original title, tests & measures, mesh word] 74

32 social web.mp. [mp=title, abstract, heading word, table of contents, key concepts, original title, tests & measures, mesh word] 162

33 social software*.mp. [mp=title, abstract, heading word, table of contents, key concepts, original title, tests & measures, mesh word] 146

34 28 or 29 or 30 or 31 or 32 or 33 56805

35 Facebook*.mp. [mp=title, abstract, heading word, table of contents, key concepts, original title, tests & measures, mesh word] 7185

36 YouTube*.mp. [mp=title, abstract, heading word, table of contents, key concepts, original title, tests & measures, mesh word] 1629

37 WhatsApp*.mp. [mp=title, abstract, heading word, table of contents, key concepts, original title, tests & measures, mesh word] 374

38 Instagram*.mp. [mp=title, abstract, heading word, table of contents, key concepts, original title, tests & measures, mesh word] 1156

39 WeChat.mp. [mp=title, abstract, heading word, table of contents, key concepts, original title, tests & measures, mesh word] 278

40 Tumblr.mp. [mp=title, abstract, heading word, table of contents, key concepts, original title, tests & measures, mesh word] 83

41 TikTok*.mp. [mp=title, abstract, heading word, table of contents, key concepts, original title, tests & measures, mesh word] 58

42 Twitter.mp. [mp=title, abstract, heading word, table of contents, key concepts, original title, tests & measures, mesh word] 3868

43 Pinterest.mp. [mp=title, abstract, heading word, table of contents, key concepts, original title, tests & measures, mesh word] 103

44 Flickr.mp. [mp=title, abstract, heading word, table of contents, key concepts, original title, tests & measures, mesh word] 137

45 Googl.mp. [mp=title, abstract, heading word, table of contents, key concepts, original title, tests & measures, mesh word] 2

46 microblog.mp. [mp=title, abstract, heading word, table of contents, key concepts, original title, tests & measures, mesh word] 137

47 podcast*.mp. [mp=title, abstract, heading word, table of contents, key concepts, original title, tests & measures, mesh word] 630

48 tweet*.mp. [mp=title, abstract, heading word, table of contents, key concepts, original title, tests & measures, mesh word] 1866

49 video sharing.mp. [mp=title, abstract, heading word, table of contents, key concepts, original title, tests & measures, mesh word] 194

50 34 or 35 or 36 or 37 or 38 or 39 or 40 or 41 or 42 or 43 or 44 or 45 or 46 or 47 or 48 or 49 60191

51 27 and 50 6265

52 limit 51 to (english language and yr="2000 - 2023") 5552

53 limit 52 to humans 5552

54 quantitative.mp. [mp=title, abstract, heading word, table of contents, key concepts, original title, tests & measures, mesh word] 115727

55 access.mp. [mp=title, abstract, heading word, table of contents, key concepts, original title, tests & measures, mesh word] 125718

56 impression*.mp. [mp=title, abstract, heading word, table of contents, key concepts, original title, tests & measures, mesh word] 35129

57 views.mp. [mp=title, abstract, heading word, table of contents, key concepts, original title, tests & measures, mesh word] 73517

58 impressions.mp. [mp=title, abstract, heading word, table of contents, key concepts, original title, tests & measures, mesh word] 11730

59 likes.mp. [mp=title, abstract, heading word, table of contents, key concepts, original title, tests & measures, mesh word] 1449

60 share.mp. [mp=title, abstract, heading word, table of contents, key concepts, original title, tests & measures, mesh word] 52761

61 comments.mp. [mp=title, abstract, heading word, table of contents, key concepts, original title, tests & measures, mesh word] 122152

62 posts.mp. [mp=title, abstract, heading word, table of contents, key concepts, original title, tests & measures, mesh word] 3890

63 HTML views.mp. [mp=title, abstract, heading word, table of contents, key concepts, original title, tests & measures, mesh word] 0

64 altmetric*.mp. [mp=title, abstract, heading word, table of contents, key concepts, original title, tests & measures, mesh word] 2

65 download*.mp. [mp=title, abstract, heading word, table of contents, key concepts, original title, tests & measures, mesh word] 2533

66 citation*.mp. [mp=title, abstract, heading word, table of contents, key concepts, original title, tests & measures, mesh word] 9110

67 in practice.mp. [mp=title, abstract, heading word, table of contents, key concepts, original title, tests & measures, mesh word] 22438

68 54 or 55 or 56 or 57 or 58 or 59 or 60 or 61 or 62 or 63 or 64 or 65 or 66 or 67 531111

69 53 and 68 1688

70 health care.m_titl. 20916

71 social care.m_titl. 942

72 "disseminat*".m_titl. 1824

73 knowledge.m_titl. 45306

74 "inform*".m_titl. 63843

75 social media.m_titl. 6369

76 "research*".m_titl. 107146

77 "evaluat*".m_titl. 89570

78 "compar*".m_titl. 100885

79 reach.m_titl. 1788

80 "engag*".m_titl. 24683

81 impact.m_titl. 77222

82 70 or 71 or 72 or 73 or 74 or 75 or 76 or 77 or 78 or 79 or 80 or 81 511055

83 53 and 82 1480

84 69 and 82 537

85 social work.mp. 35462

86 7 or 85 1099690

87 13 and 86 294306

88 15 or 16 or 17 or 18 or 19 or 20 or 21 or 22 or 23 or 24 or 25 or 26 or 87 515681

89 exp Decision Making/ or exp Nurses/ or exp Clinical Practice/ or exp Midwifery/ or exp Nursing/ or exp Allied Health Personnel/ 214147

90 88 or 89 652483

91 webcast.mp. 46

92 50 or 91 60234

93 altmetric.mp. 24

94 bibliometric.mp. or exp Bibliometrics/ 1412

95 68 or 82 or 93 or 94 971420

96 90 and 92 and 95 3635

97 limit 96 to yr="2000 - 2023" 3562

98 *Social Media/ 11367

99 97 and 98 907

EBSCO search history used for CINAHL plus, ERIC, and LISTA

S75 S73 AND S74 Expanders - Apply equivalent subjects

Search modes - Boolean/Phrase Interface - EBSCOhost Research Databases

Search Screen - Advanced Search

Database - CINAHL Plus 912

S74 (MM "Social Media") Limiters - Publication Year: 2000-2022

Expanders - Apply equivalent subjects

Search modes - Boolean/Phrase Interface - EBSCOhost Research Databases

Search Screen - Advanced Search

Database - CINAHL Plus 10,952

S73 S52 AND S70 Limiters - Publication Year: 2000-2022

Expanders - Apply equivalent subjects

Narrow by Language: - english

Search modes - Boolean/Phrase Interface - EBSCOhost Research Databases

Search Screen - Advanced Search

Database - CINAHL Plus 3,300

S72 S52 AND S70 Expanders - Apply equivalent subjects

Narrow by Language: - english

Search modes - Boolean/Phrase Interface - EBSCOhost Research Databases

Search Screen - Advanced Search

Database - CINAHL Plus 3,313

S71 S52 AND S70 Expanders - Apply equivalent subjects

Search modes - Boolean/Phrase Interface - EBSCOhost Research Databases

Search Screen - Advanced Search

Database - CINAHL Plus 3,376

S70 S53 OR S54 OR S55 OR S56 OR S57 OR S58 OR S59 OR S60 OR S61 OR S62 OR S63 OR S64 OR S65 OR S66 OR S67 OR S68 OR S69 Expanders - Apply equivalent subjects

Search modes - Boolean/Phrase Interface - EBSCOhost Research Databases

Search Screen - Advanced Search

Database - CINAHL Plus 995,809

S69 "knowledge translation" Expanders - Apply equivalent subjects

Search modes - Boolean/Phrase Interface - EBSCOhost Research Databases

Search Screen - Advanced Search

Database - CINAHL Plus 2,348

S68 "knowledge mobilisation" Expanders - Apply equivalent subjects

Search modes - Boolean/Phrase Interface - EBSCOhost Research Databases

Search Screen - Advanced Search

Database - CINAHL Plus 31

S67 (MH "Selective Dissemination of Information") OR "dissemination" Expanders - Apply equivalent subjects

Search modes - Boolean/Phrase Interface - EBSCOhost Research Databases

Search Screen - Advanced Search

Database - CINAHL Plus 15,771

S66 "in practice" Expanders - Apply equivalent subjects

Search modes - Boolean/Phrase Interface - EBSCOhost Research Databases

Search Screen - Advanced Search

Database - CINAHL Plus 681,886

S65 "download" Expanders - Apply equivalent subjects

Search modes - Boolean/Phrase Interface - EBSCOhost Research Databases

Search Screen - Advanced Search

Database - CINAHL Plus 968

S64 (MH "Citation Analysis") OR "citation" OR (MH "Bibliometrics") Expanders - Apply equivalent subjects

Search modes - Boolean/Phrase Interface - EBSCOhost Research Databases

Search Screen - Advanced Search

Database - CINAHL Plus 12,071

S63 (MH "Citation Analysis") OR "citation" OR (MH "Bibliometrics") Expanders - Apply equivalent subjects

Search modes - Boolean/Phrase Interface - EBSCOhost Research Databases

Search Screen - Advanced Search

Database - CINAHL Plus 12,071

S62 (MH "Citation Analysis") OR "citation" OR (MH "Bibliometrics") Expanders - Apply equivalent subjects

Search modes - Boolean/Phrase Interface - EBSCOhost Research Databases

Search Screen - Advanced Search

Database - CINAHL Plus 0

S61 "altmetric" Expanders - Apply equivalent subjects

Search modes - Boolean/Phrase Interface - EBSCOhost Research Databases

Search Screen - Advanced Search

Database - CINAHL Plus 148

S60 (MH "HTML") OR "HTML views" Expanders - Apply equivalent subjects

Search modes - Boolean/Phrase Interface - EBSCOhost Research Databases

Search Screen - Advanced Search

Database - CINAHL Plus 310

S59 "posts" Expanders - Apply equivalent subjects

Search modes - Boolean/Phrase Interface - EBSCOhost Research Databases

Search Screen - Advanced Search

Database - CINAHL Plus 5,252

S58 "comments" Expanders - Apply equivalent subjects

Search modes - Boolean/Phrase Interface - EBSCOhost Research Databases

Search Screen - Advanced Search

Database - CINAHL Plus 24,786

S57 "shares" Expanders - Apply equivalent subjects

Search modes - Boolean/Phrase Interface - EBSCOhost Research Databases

Search Screen - Advanced Search

Database - CINAHL Plus 7,288

S56 "likes" Expanders - Apply equivalent subjects

Search modes - Boolean/Phrase Interface - EBSCOhost Research Databases

Search Screen - Advanced Search

Database - CINAHL Plus 915

S55 "impressions" Expanders - Apply equivalent subjects

Search modes - Boolean/Phrase Interface - EBSCOhost Research Databases

Search Screen - Advanced Search

Database - CINAHL Plus 4,566

S54 "access" Expanders - Apply equivalent subjects

Search modes - Boolean/Phrase Interface - EBSCOhost Research Databases

Search Screen - Advanced Search

Database - CINAHL Plus 178,809

S53 (MH "Quantitative Studies") OR (MH "Variable") OR "quantitative" Expanders - Apply equivalent subjects

Search modes - Boolean/Phrase Interface - EBSCOhost Research Databases

Search Screen - Advanced Search

Database - CINAHL Plus 127,496

S52 S25 AND S51 Expanders - Apply equivalent subjects

Search modes - Boolean/Phrase Interface - EBSCOhost Research Databases

Search Screen - Advanced Search

Database - CINAHL Plus 8,229

S51 S32 OR S33 OR S34 OR S35 OR S36 OR S37 OR S38 OR S39 OR S40 OR S41 OR S42 OR S43 OR S44 OR S45 OR S46 OR S47 OR S48 OR S49 OR S50 Expanders - Apply equivalent subjects

Search modes - Boolean/Phrase Interface - EBSCOhost Research Databases

Search Screen - Advanced Search

Database - CINAHL Plus 42,672

S50 "video sharing" Expanders - Apply equivalent subjects

Search modes - Boolean/Phrase Interface - EBSCOhost Research Databases

Search Screen - Advanced Search

Database - CINAHL Plus 158

S49 "tweet" Expanders - Apply equivalent subjects

Search modes - Boolean/Phrase Interface - EBSCOhost Research Databases

Search Screen - Advanced Search

Database - CINAHL Plus 662

S48 (MH "Webcasts") OR "podcast" Expanders - Apply equivalent subjects

Search modes - Boolean/Phrase Interface - EBSCOhost Research Databases

Search Screen - Advanced Search

Database - CINAHL Plus 1,547

S47 "microblog" Expanders - Apply equivalent subjects

Search modes - Boolean/Phrase Interface - EBSCOhost Research Databases

Search Screen - Advanced Search

Database - CINAHL Plus 66

S46 ""googl"" Expanders - Apply equivalent subjects

Search modes - SmartText Searching Interface - EBSCOhost Research Databases

Search Screen - Advanced Search

Database - CINAHL Plus 0

S45 "googl" Expanders - Apply equivalent subjects

Search modes - Boolean/Phrase Interface - EBSCOhost Research Databases

Search Screen - Advanced Search

Database - CINAHL Plus 0

S44 "Flickr" Expanders - Apply equivalent subjects

Search modes - Boolean/Phrase Interface - EBSCOhost Research Databases

Search Screen - Advanced Search

Database - CINAHL Plus 69

S43 "Pinterest" Expanders - Apply equivalent subjects

Search modes - Boolean/Phrase Interface - EBSCOhost Research Databases

Search Screen - Advanced Search

Database - CINAHL Plus 118

S42 (MH "Twitter") Expanders - Apply equivalent subjects

Search modes - Boolean/Phrase Interface - EBSCOhost Research Databases

Search Screen - Advanced Search

Database - CINAHL Plus 970

S41 "Twitter" Expanders - Apply equivalent subjects

Search modes - Boolean/Phrase Interface - EBSCOhost Research Databases

Search Screen - Advanced Search

Database - CINAHL Plus 4,235

S40 "TikTok" Expanders - Apply equivalent subjects

Search modes - Boolean/Phrase Interface - EBSCOhost Research Databases

Search Screen - Advanced Search

Database - CINAHL Plus 125

S39 "Tumblr" Expanders - Apply equivalent subjects

Search modes - Boolean/Phrase Interface - EBSCOhost Research Databases

Search Screen - Advanced Search

Database - CINAHL Plus 57

S38 "WeChat" Expanders - Apply equivalent subjects

Search modes - Boolean/Phrase Interface - EBSCOhost Research Databases

Search Screen - Advanced Search

Database - CINAHL Plus 373

S37 (MH "Facebook") Expanders - Apply equivalent subjects

Search modes - Boolean/Phrase Interface - EBSCOhost Research Databases

Search Screen - Advanced Search

Database - CINAHL Plus 1,139

S36 (MH "Instant Messaging") OR "Instagram" Expanders - Apply equivalent subjects

Search modes - Boolean/Phrase Interface - EBSCOhost Research Databases

Search Screen - Advanced Search

Database - CINAHL Plus 1,481

S35 "WhatsApp" Expanders - Apply equivalent subjects

Search modes - Boolean/Phrase Interface - EBSCOhost Research Databases

Search Screen - Advanced Search

Database - CINAHL Plus 595

S34 "YouTube" Expanders - Apply equivalent subjects

Search modes - Boolean/Phrase Interface - EBSCOhost Research Databases

Search Screen - Advanced Search

Database - CINAHL Plus 1,562

S33 "facebook" Expanders - Apply equivalent subjects

Search modes - Boolean/Phrase Interface - EBSCOhost Research Databases

Search Screen - Advanced Search

Database - CINAHL Plus 6,202

S32 S26 OR S27 OR S28 OR S29 OR S30 OR S31 Expanders - Apply equivalent subjects

Search modes - Boolean/Phrase Interface - EBSCOhost Research Databases

Search Screen - Advanced Search

Database - CINAHL Plus 35,718

S31 "social software" Expanders - Apply equivalent subjects

Search modes - Boolean/Phrase Interface - EBSCOhost Research Databases

Search Screen - Advanced Search

Database - CINAHL Plus 56

S30 "social web" Expanders - Apply equivalent subjects

Search modes - Boolean/Phrase Interface - EBSCOhost Research Databases

Search Screen - Advanced Search

Database - CINAHL Plus 58

S29 "media sharing" Expanders - Apply equivalent subjects

Search modes - Boolean/Phrase Interface - EBSCOhost Research Databases

Search Screen - Advanced Search

Database - CINAHL Plus 40

S28 (MH "Health Information Networks") OR "open network" Expanders - Apply equivalent subjects

Search modes - Boolean/Phrase Interface - EBSCOhost Research Databases

Search Screen - Advanced Search

Database - CINAHL Plus 824

S27 (MH "Social Networking") Expanders - Apply equivalent subjects

Search modes - Boolean/Phrase Interface - EBSCOhost Research Databases

Search Screen - Advanced Search

Database - CINAHL Plus 3,338

S26 (MH "Social Media") OR "social media" Expanders - Apply equivalent subjects

Search modes - Boolean/Phrase Interface - EBSCOhost Research Databases

Search Screen - Advanced Search

Database - CINAHL Plus 32,558

S25 S14 OR S24 Expanders - Apply equivalent subjects

Search modes - Boolean/Phrase Interface - EBSCOhost Research Databases

Search Screen - Advanced Search

Database - CINAHL Plus 874,232

S24 S15 OR S16 OR S17 OR S18 OR S19 OR S20 OR S21 OR S22 OR S23 Expanders - Apply equivalent subjects

Search modes - Boolean/Phrase Interface - EBSCOhost Research Databases

Search Screen - Advanced Search

Database - CINAHL Plus 423,066

S23 (MH "Emergency Medical Technicians") OR (MH "Education, Emergency Medical Services") OR (MH "Allied Health Professions") OR (MH "Allied Health Personnel") OR "paramedic" Expanders - Apply equivalent subjects

Search modes - Boolean/Phrase Interface - EBSCOhost Research Databases

Search Screen - Advanced Search

Database - CINAHL Plus 21,335

S22 (MH "Radiologic Technologists") OR (MH "Society of Radiographers") OR (MH "Hong Kong Radiographers' Association") OR "radiographer" Expanders - Apply equivalent subjects

Search modes - Boolean/Phrase Interface - EBSCOhost Research Databases

Search Screen - Advanced Search

Database - CINAHL Plus 6,864

S21 (MH "Occupational Therapists") OR "occupational therapist" Expanders - Apply equivalent subjects

Search modes - Boolean/Phrase Interface - EBSCOhost Research Databases

Search Screen - Advanced Search

Database - CINAHL Plus 13,024

S20 (MH "Physical Therapists") OR (MH "Physical Therapist Assistants") Expanders - Apply equivalent subjects

Search modes - Boolean/Phrase Interface - EBSCOhost Research Databases

Search Screen - Advanced Search

Database - CINAHL Plus 14,703

S19 (MH "Pharmacists") Expanders - Apply equivalent subjects

Search modes - Boolean/Phrase Interface - EBSCOhost Research Databases

Search Screen - Advanced Search

Database - CINAHL Plus 18,360

S18 (MH "Social Workers") OR "social worker" Expanders - Apply equivalent subjects

Search modes - Boolean/Phrase Interface - EBSCOhost Research Databases

Search Screen - Advanced Search

Database - CINAHL Plus 13,579

S17 "doctor*" OR (MH "Physicians") Expanders - Apply equivalent subjects

Search modes - Boolean/Phrase Interface - EBSCOhost Research Databases

Search Screen - Advanced Search

Database - CINAHL Plus 139,422

S16 (MH "Midwives") OR (MH "Mental Health Personnel") OR (MH "Nurses") OR (MH "Physicians") OR (MH "Pharmacists") OR (MH "Consultants") Expanders - Apply equivalent subjects

Search modes - Boolean/Phrase Interface - EBSCOhost Research Databases

Search Screen - Advanced Search

Database - CINAHL Plus 171,746

S15 clinician* Expanders - Apply equivalent subjects

Search modes - Boolean/Phrase Interface - EBSCOhost Research Databases

Search Screen - Advanced Search

Database - CINAHL Plus 125,770

S14 S7 AND S13 Expanders - Apply equivalent subjects

Search modes - Boolean/Phrase Interface - EBSCOhost Research Databases

Search Screen - Advanced Search

Database - CINAHL Plus 546,090

S13 S8 OR S9 OR S10 OR S11 OR S12 Expanders - Apply equivalent subjects

Search modes - Boolean/Phrase Interface - EBSCOhost Research Databases

Search Screen - Advanced Search

Database - CINAHL Plus 893,547

S12 "employee" OR (MH "Rural Health Personnel") Expanders - Apply equivalent subjects

Search modes - Boolean/Phrase Interface - EBSCOhost Research Databases

Search Screen - Advanced Search

Database - CINAHL Plus 43,843

S11 (MH "Nursing Staff, Hospital") OR (MH "Staff Development") OR (MH "Medical Staff, Hospital") OR (MH "Personnel, Health Facility") OR (MH "Staff Nurses") OR (MH "Radiology Personnel") OR (MH "Health Personnel") OR (MH "Medical Staff") OR "staff" Expanders - Apply equivalent subjects

Search modes - Boolean/Phrase Interface - EBSCOhost Research Databases

Search Screen - Advanced Search

Database - CINAHL Plus 228,224

S10 "provider*" Expanders - Apply equivalent subjects

Search modes - Boolean/Phrase Interface - EBSCOhost Research Databases

Search Screen - Advanced Search

Database - CINAHL Plus 144,122

S9 "professional*" Expanders - Apply equivalent subjects

Search modes - Boolean/Phrase Interface - EBSCOhost Research Databases

Search Screen - Advanced Search

Database - CINAHL Plus 511,962

S8 "practitioner*" OR (MH "Nurse Practitioners") OR (MH "Neonatal Nurse Practitioners") OR (MH "Pediatric Nurse Practitioners") OR (MH "OB-GYN Nurse Practitioners") OR (MH "Multiskilled Health Practitioners") OR (MH "Emergency Nurse Practitioners") Expanders - Apply equivalent subjects

Search modes - Boolean/Phrase Interface - EBSCOhost Research Databases

Search Screen - Advanced Search

Database - CINAHL Plus 122,923

S7 S1 OR S2 OR S3 OR S4 OR S5 OR S6 Expanders - Apply equivalent subjects

Search modes - Boolean/Phrase Interface - EBSCOhost Research Databases

Search Screen - Advanced Search

Database - CINAHL Plus 2,560,857

S6 "Health care" Expanders - Apply equivalent subjects

Search modes - Boolean/Phrase Interface - EBSCOhost Research Databases

Search Screen - Advanced Search

Database - CINAHL Plus 598,873

S5 "social care" Expanders - Apply equivalent subjects

Search modes - Boolean/Phrase Interface - EBSCOhost Research Databases

Search Screen - Advanced Search

Database - CINAHL Plus 50,555

S4 (MH "Social Work") Expanders - Apply equivalent subjects

Search modes - Boolean/Phrase Interface - EBSCOhost Research Databases

Search Screen - Advanced Search

Database - CINAHL Plus 14,212

S3 (MH "Medical Organizations") OR (MH "Medical Practice") OR (MH "Specialties, Medical") OR "medical" Expanders - Apply equivalent subjects

Search modes - Boolean/Phrase Interface - EBSCOhost Research Databases

Search Screen - Advanced Search

Database - CINAHL Plus 769,596

S2 "health" Expanders - Apply equivalent subjects

Search modes - Boolean/Phrase Interface - EBSCOhost Research Databases

Search Screen - Advanced Search

Database - CINAHL Plus 2,075,315

S1 "health care" Expanders - Apply equivalent subjects

Search modes - Boolean/Phrase Interface - EBSCOhost Research Databases

Search Screen - Advanced Search

Database - CINAHL Plus 598,873
