# Supplementary material for: The Use of Social Media for Dissemination of Research Evidence to Health and Social Care Practitioners: Protocol for a Systematic Review
Source: JMIR Res Protoc. 2023 May 12;12:e45684. doi: 10.2196/45684 (PMC10221530; doi:10.2196/45684)
Supplement: Multimedia Appendix 2 [file resprot_v12i1e45684_app2.docx]

Appendix 2

Data extraction form

| Authors |  |
| --- | --- |
| Date |  |
| Title |  |
| Study design |  |
| Aims/research question |  |
| Summary of methods |  |
| Knowledge producer |  |
| Research evidence |  |
| Intended audience |  |
| Social media used (site, format, sharing mechanism) |  |
| Comparison groups |  |
| Controls for confounders |  |
| Outcomes collected (categorised as Reach, Engagement, Dissemination and Impact) |  |
| Results (verbatim from article) (including sample sizes per group and whole, means (S.D.) (per group), SEM, confidence intervals, medians, IQR, group or whole sample S.D., regression coefficients, difference between means (RCT), mean difference (pre/post), Cohen’s d, Hedges g etc.) |  |
| Results tables/ figures/ screenshot |  |
| Results between groups (difference between means/mean diff, S.D., effect size) |  |
| Standardised results for each group  (per social media post format) |  |
| Risk of Bias (ROB-2 or NOS star-rating, see overleaf) |  |
