# Supplementary material for: The Use of Social Media for Dissemination of Research Evidence to Health and Social Care Practitioners: Protocol for a Systematic Review
Source: JMIR Res Protoc. 2023 May 12;12:e45684. doi: 10.2196/45684 (PMC10221530; doi:10.2196/45684)
Supplement: Multimedia Appendix 3 [file resprot_v12i1e45684_app3.docx]

Appendix 3

Risk of Bias tools

Cochrane Risk of Bias tool (ROB-2)

| Bias domain and signalling question* | Response options | | |
| --- | --- | --- | --- |
|  | Lower risk of bias | Higher risk of bias | Other |
| **Bias arising from the randomisation process** | | | |
| 1.1 Was the allocation sequence random? | Y/PY | N/PN | NI |
| 1.2 Was the allocation sequence concealed until participants were enrolled and assigned to interventions? | Y/PY | N/PN | NI |
| 1.3 Did baseline differences between intervention groups suggest a problem with the randomisation process? | N/PN | Y/PY | NI |
| Risk-of-bias judgment (low/high/some concerns) |  |  |  |
| Optional: What is the predicted direction of bias arising from the randomisation process? |  | | |
| **Bias due to deviations from intended interventions** | | | |
| 2.1 Were participants aware of their assigned intervention during the trial? | N/PN | Y/PY | NI |
| 2.2 Were carers and people delivering the interventions aware of participants’ assigned intervention during the trial? | N/PN | Y/PY | NI |
| 2.3 If Y/PY/NI to 2.1 or 2.2: Were there deviations from the intended intervention that arose because of the trial context? | N/PN | Y/PY | NA/NI |
| 2.4 If Y/PY/NI to 2.3: Were these deviations likely to have affected the outcome? | N/PN | Y/PY | NA/NI |
| 2.5 If Y/PY to 2.4: Were these deviations from intended intervention balanced between groups? | Y/PY | N/PN | NA/NI |
| 2.6 Was an appropriate analysis used to estimate the effect of assignment to intervention? | Y/PY | N/PN | NI |
| 2.7 If N/PN/NI to 2.6: Was there potential for a substantial impact (on the result) of the failure to analyse participants in the group to which they were randomised? | N/PN | Y/PY | NA/NI |
| Risk-of-bias judgment (low/high/some concerns) |  |  |  |
| Optional: What is the predicted direction of bias due to deviations from intended interventions? |  | | |
| **Bias due to missing outcome data** | | | |
| 3.1 Were data for this outcome available for all, or nearly all, participants randomised? | Y/PY | N/PN | NI |
| 3.2 If N/PN/NI to 3.1: Is there evidence that the result was not biased by missing outcome data? | Y/PY | N/PN | NA |
| 3.3 If N/PN to 3.2: Could missingness in the outcome depend on its true value? | N/PN | Y/PY | NA/NI |
| 3.4 If Y/PY/NI to 3.3: Is it likely that missingness in the outcome depended on its true value? | N/PN | Y/PY | NA/NI |
| Risk-of-bias judgment (low/high/some concerns) |  |  |  |
| Optional: What is the predicted direction of bias due to missing outcome data? |  | | |
| Table continued on next page | | | |
| **Bias in measurement of the outcome** | | | |
| 4.1 Was the method of measuring the outcome inappropriate? | N/PN | Y/PY | NI |
| 4.2 Could measurement or ascertainment of the outcome have differed between intervention groups? | N/PN | Y/PY | NI |
| 4.3 If N/PN/NI to 4.1 and 4.2: Were outcome assessors aware of the intervention received by study participants? | N/PN | Y/PY | NI |
| 4.4 If Y/PY/NI to 4.3: Could assessment of the outcome have been influenced by knowledge of intervention received? | N/PN | Y/PY | NA/NI |
| 4.5 If Y/PY/NI to 4.4: Is it likely that assessment of the outcome was influenced by knowledge of intervention received? | N/PN | Y/PY | NA/NI |
| Risk-of-bias judgment (low/high/some concerns) |  |  |  |
| Optional: What is the predicted direction of bias in measurement of the outcome? |  | | |
| **Bias in selection of the reported result** | | | |
| 5.1 Were the data that produced this result analysed in accordance with a prespecified analysis plan that was finalised before unblinded outcome data were available for analysis? | Y/PY | N/PN | NI |
| Is the numerical result being assessed likely to have been selected, on the basis of the results, from: | | | |
| 5.2 ... multiple eligible outcome measurements (eg, scales, definitions, time points) within the outcome domain? | N/PN | Y/PY | NI |
| 5.3 ... multiple eligible analyses of the data? | N/PN | Y/PY | NI |
| Risk-of-bias judgment (low/high/some concerns) |  |  |  |
| Optional: What is the predicted direction bias due to selection of the reported results? |  | | |
| **Overall bias** | | | |
| Risk-of-bias judgment (low/high/some concerns) |  |  |  |
| Optional: What is the overall predicted direction of bias for this outcome? |  | | |
| Table legend: *Signalling questions for bias due to deviations from intended interventions relate to the effect of assignment to intervention  Y=yes; PY=probably yes; PN=probably no; N=no; NA=not applicable; NI=no information. | | | |

NEWCASTLE - OTTAWA QUALITY ASSESSMENT SCALE

Note: A study can be awarded a maximum of one star for each numbered item within the Selection and Exposure categories. A maximum of two stars can be given for Comparability.

CASE CONTROL STUDIES

| Selection | 1) Is the case definition adequate? | a) yes, with independent validation * |  |
| --- | --- | --- | --- |
|  |  | b) yes, e.g., record linkage or based on self-reports |  |
|  |  | c) no description |  |
|  | 2) Representativeness of the cases | a) consecutive or obviously representative series of cases * |  |
|  |  | b) potential for selection biases or not stated |  |
|  | 3) Selection of Controls | a) community controls * |  |
|  |  | b) hospital controls |  |
|  |  | c) no description |  |
|  | 4) Definition of Controls | a) no history of disease (endpoint) * |  |
|  |  | b) no description of source |  |
| Comparability | 1) Comparability of cases and controls on the basis of the design or analysis | a) study controls for _______________ (Select the most important factor.) * |  |
|  |  | b) study controls for any additional factor * |  |
| (This criterion could be modified to indicate specific control for a second important factor.) | | |  |
| Exposure | 1) Ascertainment of exposure | a) secure record (e.g., surgical records) * |  |
|  |  | b) structured interview where blind to case/control status * |  |
|  |  | c) interview not blinded to case/control status |  |
|  |  | d) written self-report or medical record only |  |
|  |  | e) no description |  |
|  | 2) Same method of ascertainment for cases and controls | a) yes * |  |
|  |  | b) no |  |
|  | 3) Non-Response rate | a) same rate for both groups * |  |
|  |  | b) non respondents described |  |
|  |  | c) rate different and no designation |  |

COHORT STUDIES

| Selection | 1) Representativeness of the exposed cohort | a) truly representative of the average _______________ (describe) in the community * |  |
| --- | --- | --- | --- |
|  |  | b) somewhat representative of the average ______________ in the community * |  |
|  |  | c) selected group of users e.g., nurses, volunteers |  |
|  |  | d) no description of the derivation of the cohort |  |
|  | 2) Selection of the non-exposed cohort | a) drawn from the same community as the exposed cohort * |  |
|  |  | b) drawn from a different source |  |
|  |  | c) no description of the derivation of the non-exposed cohort |  |
|  | 3) Ascertainment of exposure | a) secure record (e.g., surgical records) * |  |
|  |  | b) structured interview * |  |
|  |  | c) written self-report |  |
|  |  | d) no description |  |
|  | 4) Demonstration that outcome of interest was not present at start of study | a) yes * |  |
|  |  | b) no |  |
| Comparability | 1) Comparability of cohorts on the basis of the design or analysis | a) study controls for _____________ (select the most important factor) * |  |
|  |  | b) study controls for any additional factor * |  |
| (This criterion could be modified to indicate specific control for a second important factor.) | | |  |
| Outcome | 1) Assessment of outcome | a) independent blind assessment * |  |
|  |  | b) record linkage * |  |
|  |  | c) self-report |  |
|  |  | d) no description |  |
|  | 2) Was follow-up long enough for outcomes to occur | a) yes (select an adequate follow up period for outcome of interest) * |  |
|  |  | b) no |  |
|  | 3) Adequacy of follow up of cohorts | a) complete follow up - all subjects accounted for * |  |
|  |  | b) subjects lost to follow up unlikely to introduce bias - small number lost - > ____ % (select an adequate %) follow up, or description provided of those lost) * |  |
|  |  | c) follow up rate < ____% (select an adequate %) and no description of those lost |  |
|  |  | d) no statement |  |
